# Robot-assisted fluorescent sentinel lymph node identification in early-stage colon cancer

**DOI:** 10.1007/s00464-023-10394-2

**Published:** 2023-09-18

**Authors:** Daan J. Sikkenk, Andrea J. Sterkenburg, Thijs A. Burghgraef, Halil Akol, Matthijs P. Schwartz, René Arensman, Paul M. Verheijen, Wouter B. Nagengast, Esther C. J. Consten

**Affiliations:** 1 Department of Surgery, University of Groningen, University Medical Center Groningen, Hanzeplein 1, 9713 GZ Groningen, The Netherlands; 2 Department of Surgery, Meander Medical Center, Maatweg 3, 3813 TZ Amersfoort, The Netherlands; 3 Department of Gastroenterology and Hepatology, University of Groningen, University Medical Center Groningen, Hanzeplein 1, 9713 GZ Groningen, The Netherlands; 4 Department of Gastroenterology, Meander Medical Center, Maatweg 3, 3813 TZ Amersfoort, The Netherlands; 5 Department of Pathology, Meander Medical Center, Maatweg 3, 3813 TZ Amersfoort, The Netherlands

**Keywords:** Colon cancer, Sentinel lymph node (SLN), Indocyanine green (ICG), Near-infrared fluorescence (NIRF), Image-guided surgery, Robotic surgical procedures

## Abstract

**Background:**

Patients with cT1-2 colon cancer (CC) have a 10–20% risk of lymph node metastases. Sentinel lymph node identification (SLNi) could improve staging and reduce morbidity in future organ-preserving CC surgery. This pilot study aimed to assess safety and feasibility of robot-assisted fluorescence-guided SLNi using submucosally injected indocyanine green (ICG) in patients with cT1-2N0M0 CC.

**Methods:**

Ten consecutive patients with cT1-2N0M0 CC were included in this prospective feasibility study. Intraoperative submucosal, peritumoral injection of ICG was performed during a colonoscopy. Subsequently, the near-infrared fluorescence ‘Firefly’ mode of the da Vinci Xi robotic surgical system was used for SLNi. SLNs were marked with a suture, after which a segmental colectomy was performed. The SLN was postoperatively ultrastaged using serial slicing and immunohistochemistry, in addition to the standard pathological examination of the specimen. Colonoscopy time, detection time (time from ICG injection to first SLNi), and total SLNi time were measured (time from the start of colonoscopy to start of segmental resection). Intraoperative, postoperative, and pathological outcomes were registered.

**Results:**

In all patients, at least one SLN was identified (mean 2.3 SLNs, SLN diameter range 1–13 mm). No tracer-related adverse events were noted. Median colonoscopy time was 12 min, detection time was 6 min, and total SLNi time was 30.5 min. Two patients had lymph node metastases present in the SLN, and there were no patients with false negative SLNs. No patient was upstaged due to ultrastaging of the SLN after an initial negative standard pathological examination. Half of the patients unexpectedly had pT3 tumours.

**Conclusions:**

Robot-assisted fluorescence-guided SLNi using submucosally injected ICG in ten patients with cT1-2N0M0 CC was safe and feasible. SLNi was performed in an acceptable timespan and SLNs down to 1 mm were detected. All lymph node metastases would have been detected if SLN biopsy had been performed.

Colorectal cancer is the 3rd most common malignancy in the Netherlands and the second leading cause of cancer-related deaths [1, 2]. More local excisions and polypectomies are performed due to an increased incidence of T1-2 tumours following population screening for colorectal cancer [3]. However, local excisions are limited by the inability to assess lymph node status, which is one of the most important reasons for secondary surgery and adjuvant chemotherapy. Although the risk of lymph node metastases is relatively low, namely 10–20% in pT1-2 colon cancer (CC) [4, 5], segmental resections are routinely performed. However, segmental resections result in substantial morbidity [6], mortality (2.4–3.1%) [6–8], hospitalisation [6], and reduction in quality of life (e.g., as a consequence of stomas) [9, 10]. Another drawback is that up to 20% of patients with stage I and II CC still develop recurrent disease or distant metastases despite radical segmental resection [11–13], which could result from insufficient sensitivity of conventional histopathological examination for (micro)metastases [12, 14].

Sentinel lymph node (SLN) biopsy combined with local treatment of early-stage CC could potentially be sufficient for therapy and staging, resulting in reduced morbidity due to organ-preserving surgery [15, 16]. Furthermore, SLN biopsy has been proposed to improve detection of lymph node metastases, due to ultrastaging techniques [12, 14]. Several studies regarding SLN identification (SLNi) in CC have been published. Although the technique might be applicable in clinical practice, relatively low sensitivity rates were found, varying between 70 and 76% with subsequent high rates of false negatives [17–19]. However, it is suggested that using near-infrared fluorescence (NIRF), optimising the tracer injection method, and patient selection could improve the outcomes of SLNi [20]. Especially SLNi in patients with cT1-2 tumours after submucosal peritumoral tracer injection might lead to a better sensitivity [20]. Furthermore, we hypothesised that the stable, magnified 3D view of the da Vinci Xi could improve identification of SLNs. This pilot study aimed to assess safety and feasibility of robot-assisted fluorescence-guided SLNi in ten patients with cT1-2N0 CC using intraoperative submucosal injection of indocyanine green (ICG).

## Materials and methods

### Study design

This prospective pilot study assessed safety and feasibility of robot-assisted SLNi using submucosal, peritumoral injection of ICG in patients with cT1-2N0M0 CC. After SLNi, segmental colon resection was performed according to standard of care.

Ten consecutive patients were identified from multidisciplinary team meetings at the large teaching hospital Meander Medical Center (the Netherlands). Preoperative work-up included colonoscopy (with biopsy) and thoracoabdominal computed tomography according to current guidelines [21]. The study was approved by the local medical ethics committee (MEC-U, Nieuwegein, the Netherlands, NL71065.100.19) and registered at https://trialsearch.who.int/ (formerly registered at www.trialregister.nl, NL8901) before inclusion of the first patient.

### Patient population

Eligibility criteria for participation were: (1) cT1-2N0M0 colon cancer, (2) age ≥ 18 years, (3) and provided oral and written informed consent. Exclusion criteria were: (1) prior local excision of the tumour, (2) suspicion of lymph node or distant metastases, (3) contraindication for robot-assisted surgery or the use of ICG (i.e., iodine allergy, severe kidney or liver failure, hyperthyroidism or an autonomously functioning thyroid adenoma), (4) pregnancy and lactation, (5) a tumour too large to pass endoscopically, (6) and metastatic disease or T4 tumour discovered during intraoperative staging. Patients underwent mechanical bowel preparation according to local hospital protocol for the intraoperative colonoscopy.

### Outcomes

The primary outcomes were: (1) SLNi rate (defined as the number of patients with one or more SLNs identified divided by total number of patients) and (2) rate of adverse events related to ICG injection (defined as the number of adverse events related to injection ICG divided by number of procedures). Secondary outcomes included: total number of SLNs, false negative SLNs, true negative SLNs, sensitivity, upstaging, aberrant lymph nodes, accuracy, and negative predictive value. Furthermore, intraoperative times were noted for the different steps of the procedure.

### Technique

SLNi was performed using the da Vinci Xi (Intuitive Surgical Inc., Sunnyvale, USA) robot-assisted surgical platform. First, the abdominal cavity was inspected to rule out a T4 tumour or visible metastases that would exclude the patient from continuing the study. Second, 25 mg of ICG (Verdye, Diagnostic Green GmbH, Aschheim-Dornach, Germany) was diluted in 5 ml water for injection. Third, a gastroenterologist performed a colonoscopy and submucosally injected 1 ml of ICG in four aliquots around the tumour (4 ml in total). After the first four patients, aliquots were created with 0.9% NaCl. Subsequently, the ICG was injected in this aliquot to minimise risk of intra-abdominal spillage of ICG. After injection of ICG, the mesocolon was inspected with the NIRF ‘Firefly’ mode of the da Vinci Xi. Thereafter, SLNs were marked with a suture. Afterwards, segmental resection was performed according to standard of care to ensure removal of lymph node metastases in non-SLNs. If no sentinel lymph nodes were found during in vivo examination, a repeated ex vivo fluorescence examination of the specimen was performed using the Firefly.

### Pathological examination of the lymph nodes

Immediately after surgery, the pathologist used the sutures to identify and isolate SLNs from the specimen. Excised SLNs were again evaluated for the presence of fluorescence using the Firefly. SLNs were ultrastaged: paraffin-embedded tissue blocks were sectioned at 250 μm intervals and examined at five levels using hematoxylin–eosin staining and immunohistochemically staining with a cytokeratin AE1/AE3 antibody cocktail, based on the current standard pathology assessment of SLNs in breast cancer [22]. Non-SLNs were stained with hematoxylin–eosin. In case both the SLNs and non-SLNs were negative for metastases after initial assessment, all non-SLNs were also ultrastaged.

### Definitions

Three categories of lymph node metastases were defined: isolated tumour cells (≤ 0.2 mm), micrometastases (0.2–2 mm), and macrometastases (> 2 mm). Micrometastases and macrometastases were considered positive lymph nodes. SLNs were defined as the first, up to four, fluorescent lymph nodes visible after injection. Other lymph nodes were defined as non-SLNs. Upstaging was defined as patients with lymph nodes metastases after ultrastaging, and aberrant lymph nodes as SLNs outside the planned resection margins. False negative SLNs were defined as negative SLNs with positive non-SLNs, and true negative SLNs as the SLN and the non-SLN not containing metastases. Sensitivity was the proportion of patients with a positive SLN divided by the total number of patients with metastases (the sum of patients with a positive SLN and false negative SLNs). Accuracy was the sum of positive SLNs and true negative SLNs as a percentage of patients in which an SLN was found. Preoperative cT-classification was based on computed tomography. If a tumour could not be localised (cTx), it was considered cT1-2. *Colonoscopy time* was defined as the time from start of colonoscopy until the end of the colonoscopy. *Detection time* was the time from first ICG injection to first SLNi. Total *SLNi time* was the time from start of the colonoscopy to continuing the surgical procedure according to standard of care, and *operation time* was the time from first incision to wound closure.

### Statistics

Descriptive statistics were used to describe patient and tumour characteristics.

## Results

### Patients

Ten consecutive patients with cT1-2N0M0 CC underwent curative resection between September 2020 and August 2021. Seven males and three females were included with a mean age of 70 years (range 59–84 years), body mass index ranged from 21 to 35 kg/m^2^, and 60% of patients had ASA 2 score (Table 1).

**Table 1:** Demographic data of patients

Baseline characteristic	*N*
Sex	
Female	3
Male	7
Age, mean (SD), years	69.3 (8.1)
BMI, median (range), kg/m^2^	27 [21–35]
ASA score	
1	1
2	6
3	2
4	1

*ASA* American Society of Anesthesiology, *BMI* body mass index, *SD* standard deviation

### Intraoperative outcomes

**Fig. 1:**
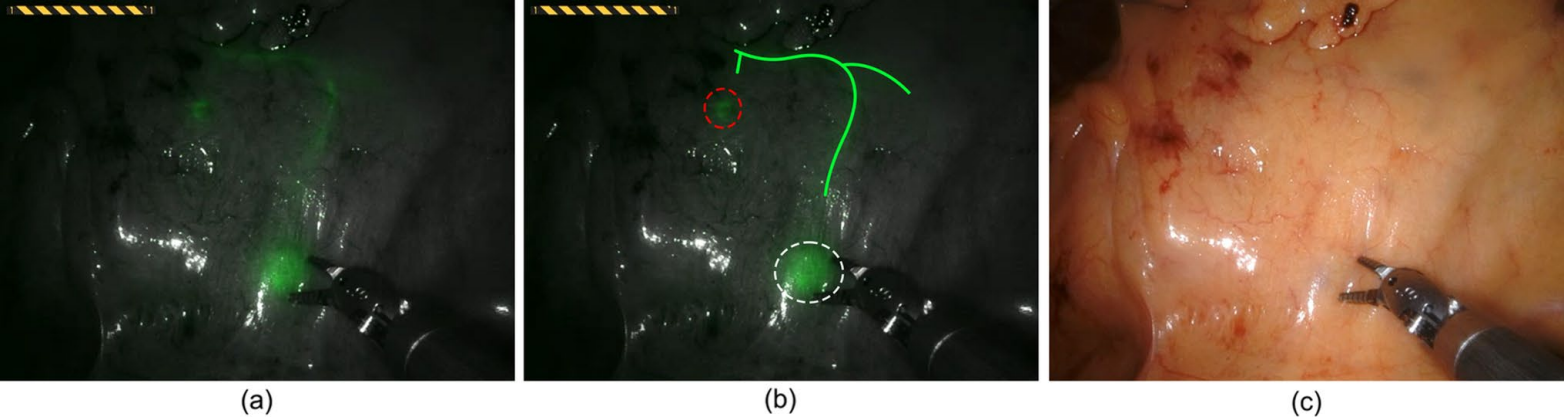
**a** The near-infrared fluorescence (NIRF) view of the mesocolon, **b** annotated picture, and **c** and the white-light picture of the same view as a reference. **a** Two sentinel lymph nodes (SLNs) and the corresponding lymphatic vessels were visible using NIRF. **b** The first and second visible SLN are highlighted with a white and red circle, respectively; both SLNs were to become brighter after waiting some time. The lymph vessels are highlighted in green. Bringing the camera closer to the tissue would also improve visibility of the SLNs (Color figure online)

There were no adverse events related to ICG or its injection. Minimal intra-abdominal leakage of ICG occurred in patient 4 (Table 2). Thereafter, 0.9% NaCl submucosal aliquots were first created before ICG administration; still another leakage occurred in patient 9. Nevertheless, SLNi was possible regardless of minimal intra-abdominal leakage of ICG in the two patients. In total, 22 sutures were placed to mark a potential SLN (Fig. 1). One suture turned out to be an injection spot during ex vivo examination, but two other sutures correctly marked SLNs in this patient. SLNi rate was 100%; at least one SLN (mean 2.3 SLNs per patient) was identified in all patients (Table 3). In patient 5 and 10, two fluorescent lymph nodes were found near the same suture, thus both were assigned as SLN. In patient 7, a limited fluorescent SLN was visible, and due to uncertainty of having marked an SLN, ex vivo fluorescence examination revealed an additional SLN. No aberrant SLNs were found.

**Table 2:** Procedural details

Patient	Location	cT-stage	Colonoscopy time	Detection time	Total SLNi time	Operation time
1	Splenic flexure	cT1-2	42	17	75	240
2	Ascending	cT2	12	6	35	214
3	Splenic flexure	cT2	44	4	48	164
4	Caecum	cT2	14	21	30	124
5	Sigmoid	cT1-2	9	6	18	117
6	Sigmoid	cT1-2	12	2	29	92
7	Sigmoid	cT2	11	54	39	132
8	Ascending	cT1-2	15	3	31	133
9	Sigmoid	cT2	8	7	28	123
10	Sigmoid	cT1-2	10	6	27	148

*SLNi* sentinel lymph node identification

**Table 3:** Pathology report

Patient	Histology	No. of SLNs	SLN diameter (mm)^a^	No. of non-SLNs	No. of metastatic lymph nodes	pTN
1	Adeno	1	3	14	0	pT1N0
2	Mucinous	3	3, 11, 12	42	2 (2 metastatic SLNs)^b^	pT3N1
3	Mucinous	1	12	11	0	pT2N0
4	Mucinous	1	4	26	0	pT3N0
5	Adeno	2 (+ 1)^c^	5, 6, 6	13	0	pT2N0
6	Adeno	3	6, 1, 8	19	0	pT3N0
7	Adeno	2	9, 9	10	5 (2 metastatic SLNs)	pT3N2a
8	Adeno	2	3, 3	41	0	pT3N0
9	Adeno	4	2, 5, 9, 8	8	0	pT2N0
10	Adeno	2 (+ 1)^c^	5, 2, 13	10	0	pT1N0

*Adeno* adenocarcinoma, *Mucinous* mucinous adenocarcinoma, *SLN* sentinel lymph node

^a^Size of consecutive SLNs

^b^One SLN contained macrometastases and one SLN contained isolated tumour cells

^c^Two SLNs were found near a single suture

The median colonoscopy time was 12 min (range 8–44 min), detection time 6 min (range 2–54 min), total SLNi time 30.5 min (range 18–75 min), and operation time 133 min (range 92–240 min). In two patients, conversion was required due to limited overview; SLNi was already completed in one patient. In the other patient SLNi was performed after laparotomy by covering the abdominal cavity with gauzes, dimming the operating room lights, and inspecting the mesocolon whilst holding the da Vinci camera. In addition, one conversion was required due to bleeding during the segmental dissection phase and postoperative blood transfusion was required. Subsequently, the patient experienced postoperative ileus and wound infection, which later resolved.

### Pathological examination

**Fig. 2:**
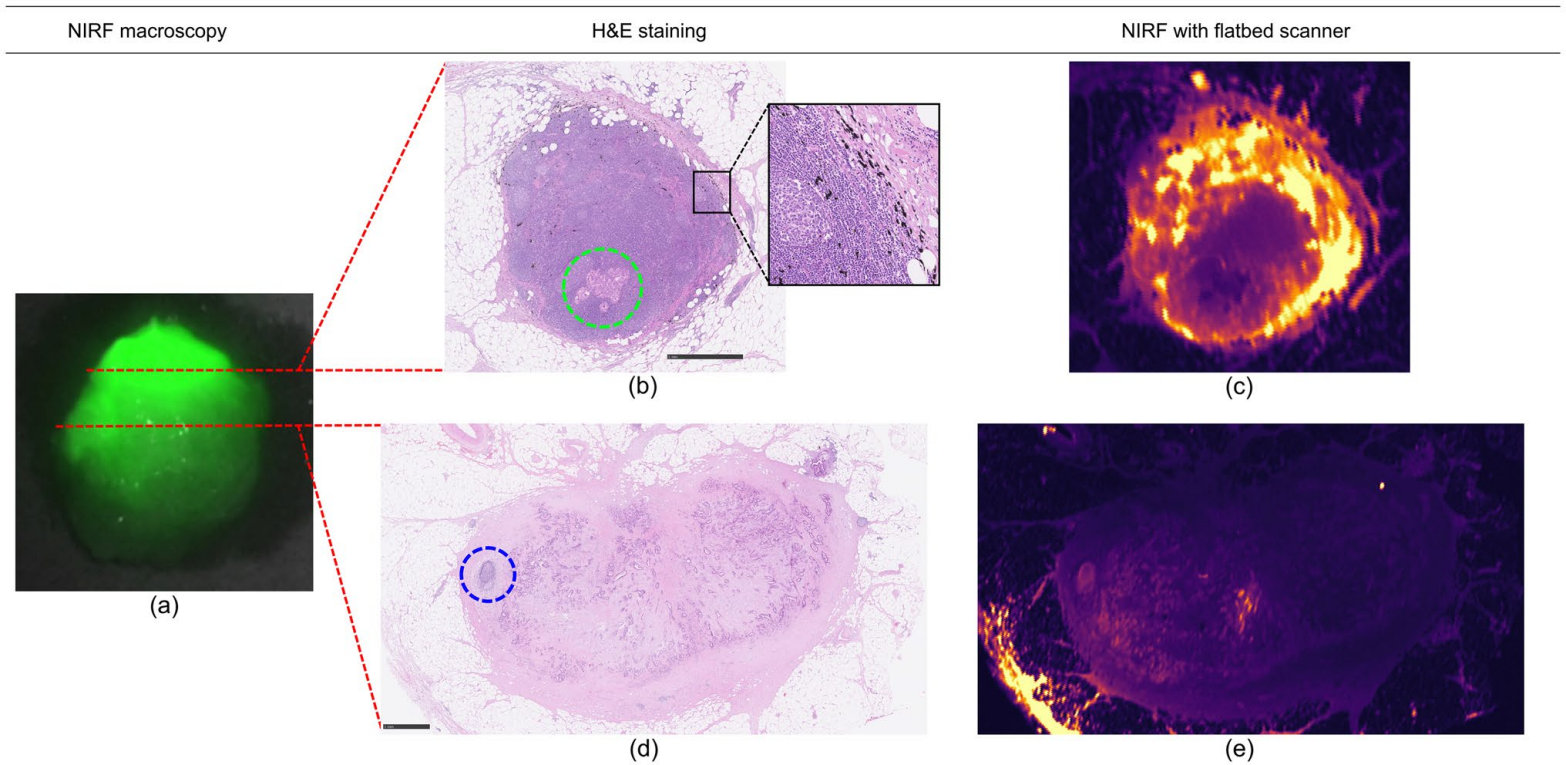
Massive lymph node metastases in a SLN. **a** Ex vivo macroscopic view with the Firefly after the SLN (9 mm in diameter) was isolated from the specimen. NIRF in the SLN was unevenly distributed. **b**, **d** Two intervals of the same SLN on microscope slides with hematoxylin and eosin (H&E) staining with a black bar demonstrating 1 mm. **c**, **e** The NIRF view of the same slides with the LI-COR Odyssey CLx flatbed imager (LI-COR Biosciences Inc., NE, USA); bright yellow represents a high fluorescence signal, while dark purple represents a low signal. **b** Lymph node metastases, displayed in the green circle, surrounded by normal lymph node tissue. Sidenote: ink from the endoscopic tattoo was visible in the SLN; an example is magnified. **c** The flatbed scanner showed reduced NIRF in and near the lymph node metastases. **d** Metastases almost entirely occupied this SLN interval with only a small area of residual normal lymph node tissue displayed in the blue circle. **e** The NIRF signal is low to absent in the microscope slide compared to panel (**c**) (Color figure online)

Presence of fluorescence was confirmed in all SLNs after isolating the SLN from the specimen. Two patients, patient 2 and 7, had macrometastases in the SLN (Table 3). In addition, isolated tumour cells were found in a second SLN of patient 2. In patient 7, two SLNs were identified although the SLNs showed reduced NIRF. These two SLNs contained massive lymph node metastases with little residual lymph node tissue. Microscope slides of massive lymph node metastases showed that fluorescence is (largely) absent in and around metastases compared to normal lymph node tissue (Fig. 2). Furthermore, there were no patients with false negative SLNs. Thus, eight patients had a true negative SLN (Table 3). As a result, sensitivity and accuracy were 100%, and the negative predictive value was 1. The diameter of SLNs ranged from 1 to 13 mm. No patient was upstaged after ultrastaging. Five out of ten patients had a pT3 tumour.

## Discussion

This pilot study aimed to assess safety and feasibility of robot-assisted fluorescence-guided SLNi using submucosally injected ICG in patients with cT1-2N0 CC. At least one SLN was identified in every patient (median 2.3 SLNs). The SLNs correctly represented the presence or absence of lymph node metastases in all patients. No tracer (injection-)related adverse events occurred. One intraoperative bleeding occurred during the segmental dissection phase of the procedure, after having completed SLNi, which required a laparotomy and blood transfusion.

Twenty-one sutures correctly marked an SLN, although a single suture incorrectly marked an injection spot as an SLN. Similarly, Currie et al. described that in 2 out of 30 patients a fluorescent marked spot was not a lymph node [23]. Performing white-light examination during SLNi, thus turning off NIRF mode, might prevent incorrectly marking an injection spot. Presence of fluorescence was confirmed in all SLNs after isolating the SLN from the specimen. Since all lymph node metastases were detected with SLNi, sensitivity and accuracy were 100% in this pilot study. In a meta-analysis (*N* = 139), SLNi for T1-2 CC had 80% sensitivity and 98% accuracy [20], which might already be sufficient for SLNi when considering the risk of lymph node metastases is 10–20% for pT1-2 tumours [4, 5]. The negative consequences of missed lymph node metastases could outweigh the morbidity and mortality of segmental resection [6–10]. Ultimately, to prove the reliability of SLNi a study of 3000 patients would be required (assuming a 10% chance of lymph node metastases, 95% identification rate, and 5% false negative rate) [24], which might not be feasible.

In one patient, two SLNs with massive lymph node metastases were identified with the Firefly although the SLNs had a (subjectively) low NIRF intensity compared to other SLNs. The massive lymph node metastases, with little residual lymph node tissue, might have resulted in limited ICG uptake in these SLNs. The effect of massive lymph node metastases on tracer uptake was also seen by Ribero et al. in 1/70 patients following ICG lymphadenectomy [25], but was also reported in earlier studies for locally advanced tumours and lymph node metastases that blocked lymphatic vessels [26, 27]. NIRF is negatively correlated with the percentage of lymph node that is occupied with metastases, resulting in that no fluorescence was detectable in lymph nodes containing > 90% cancer cells [28]. Thus, one should be aware that limited, or no, visible SLNs could signify the presence of lymph node metastases. Therefore, ex vivo SLNi should be attempted to find additional SLNs in case of uncertainty or standard segmental resection should be performed. Ideally, contrast between SLNs and background should be high to facilitate identification. SLNi can be hindered by background NIRF (i.e., scattering, autofluorescence of tissue, and automatic gain of NIRF systems [29–33]) and spillage of ICG [34]. In our study spillage occurred two times; once after the introduction of 0.9% NaCl aliquots before ICG injection. After the NaCl injection failed to form a proper aliquot, indicating a possible transmural penetration, changing the injection site could have prevented the second spillage of ICG. However, SLNi was still possible in both cases of spillage. Spillage may occur in both subserosal and submucosal injection methods [20], however, it is hypothesised that submucosal administration could lead to less false negative SLNs since it is injected near the tumour [35].

Although several methods of injection of ICG are being used, we chose to perform intraoperative injection of ICG for SLNi as more, and higher echelon, lymph nodes will be visible after prolonged waiting. Median detection time of SLNs was 6 min. A systematic review found that more SLNs were identified 15 min after injection [35]. Cahill et al. had similar laparoscopic SLN detection times after intraoperative ICG injection, but also demonstrated that preoperative injection of ICG led to detection of 12 fluorescent lymph nodes [36]. In addition, the short interval between injection of ICG and SLNi could be why no aberrant lymph nodes were observed in our study. For instance, 49% of patients had aberrant lymph nodes after ICG was injected 24 h preoperatively [25]. However, Saha et al. demonstrated that only 1% of patients had isolated metastases in aberrant lymph nodes, which were all found in T3-4 tumours [37]. In another study, Saha et al. showed that all lymph node metastases were found in the segment of the colon with the primary tumour while no metastases were found in the distal segment of the colon (outside the field of SLNs) if the proximal part was negative [38]. Therefore, we question the importance of aberrant SLNs in T1-2 CC.

Both patient and tumour characteristics were important for SLNi. Colonoscopy time and total SLNi time were substantially longer in two patients with a tumour in the splenic flexure. In one of these patients, the tumour was preoperatively localised in the descending colon and ultimately re-docking of the robot was required. In the other patient, conversion to laparotomy was required due to a limited view associated with obesity. After laparotomy, SLNi could still be performed by covering the abdominal cavity with gauzes and dimming lights in the operating theatre. Injection of ICG was completed within 15 min in the remaining eight patients. Regarding tumour characteristics, this study intended to include only patients with pT1-2 tumours as more invasive tumours might alter lymph drainage patterns [36, 39–46]. Six prior studies distinguished between SLNi in patients with T1-2 tumours and T3-4 tumours [23, 35, 41, 43, 45, 47]. However, only two studies had false negative SLNs, which used subserosal injection or older camera systems [23, 41]. More sensitive NIRF laparoscopes have been developed in recent years, and a stable robotic surgical platform with magnified 3D view could be highly beneficial for detecting small SLNs; even down to 1 mm as shown in our study. As reported in literature, half of positive lymph nodes are present in ≤ 5–6 mm lymph nodes and the largest metastatic lymph node might only be 2–3 mm [48, 49]. Therefore, the da Vinci Xi robot would be capable enough to detect clinically relevant SLNs using the SLNi technique described in this study. Regarding patient selection based on tumour characteristics, 50% of patients in this study had pT3 tumours despite complete preoperative work-up according to guidelines. Likewise, Currie et al. intended to perform SLNi in patients with T1-2 tumours: 13 out of 27 patients had pT3 tumours. The number of patients incorrectly preoperatively staged as cT1-2 corresponds to the 43% understaging of cT1-2 patients found in our previously published study that was conducted as a result of this pilot study [50]. More research should be performed regarding preoperative staging of CC for patient-tailored treatment. Nevertheless, SLNi in our study was not negatively impacted by patients with a pT3 tumours.

### Strengths and limitations

To our knowledge, this is the first study exploring robot-assisted SLNi using intraoperative submucosal ICG injections in ten patients, as prior studies on robot-assisted SLNi used preoperative ICG injection for lymphadenectomy [25, 51, 52]. Consecutive patients were asked to participate and neither BMI nor tumour location were exclusion criteria. Robot-assisted segmental resection of a tumour in the splenic flexure and high BMI was more challenging. Thus, a more careful selection of patients might aid SLNi as a good overview of the mesocolon is required, but difficult (patient) characteristics ultimately did not prevent SLNi.

Although colonoscopy time was relatively short, intraoperative colonoscopies were performed by a gastroenterologist, which required logistical coordination and downtime was not measured. SLNi cases were scheduled first of the day and resulted in shorter downtime for the gastroenterologist. In other hospitals, surgeons might inject ICG themselves. Alternatively, tumour-targeted tracers could improve logistics as preoperative administration is possible. Bevacizumab-800CW [53, 54], cetuximab-800CW [55, 56] and SGM-101 [57], targeting, respectively, VEGF-A, EGFR, and CEA, are currently being investigated for the detection of resection margins of the primary tumour and (lymph node) metastases. More importantly, tumour-targeted tracers can be intravenously administered to detect lymph node metastases and would eliminate the need for colonoscopy. In addition, intravenous administration is not hindered by massive lymph node metastases or locally advanced cancers that block lymph flow.

## Conclusion

Robot-assisted fluorescence-guided SLNi in cT1-2N0M0 CC using intraoperative submucosal ICG injection was safe, feasible, and was performed in a timely manner. SLNi was achieved in all ten patients and SLNs down to 1 mm were detected. Furthermore, SLNi correctly identified the presence of lymph node metastases if SLN biopsy had been performed instead of segmental resection. Future studies in SLN biopsy and tumour-targeted tracers for the detection of lymph node metastases in CC are warranted.

## Abbreviations

CC: Colon cancer
ICG: Indocyanine green
NIRF: Near-infrared fluorescence
SLN: Sentinel lymph node
SLNi: Sentinel lymph node identification
cTNM: Clinical TNM stage
pTNM: Pathological TNM stage
